# The Experience of Succeeding and Failing at Self-Control: A Qualitative Analysis

**DOI:** 10.3389/fpsyg.2022.774059

**Published:** 2022-04-06

**Authors:** Fernanda C. Andrade, Rick H. Hoyle

**Affiliations:** Department of Psychology and Neuroscience, Duke University, Durham, NC, United States

**Keywords:** self-control, emotion, goals, self-regulation, inhibition, initiation, motivation

## Abstract

Despite the importance of emotions for learning and performance of future behaviors, few studies have attempted to qualitatively describe emotions that arise in response to self-control successes and failures. This study is the first qualitative analysis to examine the complexity of goals that give rise to self-control challenges of two types—initiation and inhibition—and the emotions that follow success and failure experiences. Thematic analysis revealed a sometimes blurred line between inhibition and initiation, and a variety of goals that challenge views that successful self-control is good and unsuccessful self-control is bad. Descriptions of self-control challenges and resulting experiences further uncovered distinctions and apparent emotional profiles characteristic of self-control dilemmas involving inhibition or initiation, suggesting that these two forms of self-control are not only theoretically but also experientially distinct.

## Introduction

In pursuing any goal, people likely encounter situations in which their pursuit clashes with another important goal. Common examples are eating indulgent foods while dieting, and continuing to work on an important yet boring task. Situations such as these, in which two goals conflict with one another, call for self-control. Self-control refers to decisions or behaviors that are consistent with a given goal despite other concurrent yet conflicting goals or values (Fujita, 2011; Hoyle and Davisson, 2016). In the dieting example, good self-control would mean resisting the temptation to eat indulgent foods while on a diet; not only when the desire arises, but also for the duration of the desire.

Self-control is most commonly understood as the inhibition of goal-inconsistent impulses. More recently, researchers have noted that self-control can also involve initiation of desired behaviors despite challenges to doing so. Self-control can, for example, involve inhibiting the temptation to eat a desirable food to comply with a diet, but also involve initiating the act of eating a less desirable food to comply with a dieting goal. These two types of self-control have been referred to by different names, but all refer to the idea that self-control can involve engaging in desired behaviors and foregoing undesired ones. A few such names are start and stop control (de Boer et al., 2011), initiatory and inhibitory control (de Ridder et al., 2012), and self-control by initiation and inhibition (Hoyle and Davisson, 2016).

The literature is rich with evidence of the benefits of good self-control, including psychological well-being and engagement in health promoting behaviors (de Ridder et al., 2012). However, because self-control involves a conflict between desired or valued goals, it is perhaps unsurprising that poor self-control and indulging in a desired behavior can be a pleasant experience—after all, the failure is still met with a reward (Tice and Bratslavsky, 2000). At the same time, because one valued goal is foregone in favor of another, poor self-control can also be related to negative emotions such as guilt and regret, which can in turn dampen the pleasure of indulgence (Hofmann et al., 2013; Kotabe et al., 2019). The opposite is true of good self-control: Although self-control successes are often accompanied by feelings of pride (Hofmann et al., 2013), factors like how conflicted people feel during a dilemma can influence how satisfied and proud they feel about their success (Becker et al., 2019).

The positive and negative emotions that accompany self-control successes and failures have important consequences for learning and motivating future behaviors by reinforcing or discouraging hedonic choices (Chun et al., 2007; Keinan and Kivetz, 2008; Onwezen et al., 2014; Etkin et al., 2015). For instance, the expectation that self-control failures will engender negative emotions like guilt and shame can discourage self-control failures (Patrick et al., 2009; Kotabe et al., 2019). Emotional consequences of self-control decisions can also foster future success by increasing awareness of decisional conflicts (Hoffman and Fisher, 2012), and motivating engagement in actions to repair mishaps (Amodio et al., 2007). Moreover, emotions arising from self-control successes and failures can alter the value of goals, ultimately influencing the likelihood of exerting future self-control (Hoffman and Fisher, 2012). Such consequences could have important implications for why people do things they know they should not do, or fail to do things they know they should—failures of self-control can feel good and successes may feel unsatisfying.

Despite the importance of emotions for learning and performance of future behaviors (Patrick et al., 2009; Hoffman and Fisher, 2012; Etkin et al., 2015), few studies have attempted to qualitatively describe emotions that arise in response to the self-control successes and failures. In one of the first studies to examine this link, Giner-Sorolla (2011) asked participants to generate emotions that they expected to experience after succeeding or failing to exert self-control. This seminal work was among the first to show that self-control failures in the form of indulgences can feel good and inhibitory successes can feel unsatisfying, as evidenced by participants’ mentions of both positive and negative emotions in response to successes and failures of self-control. More recently, studies using methodologies like ecological momentary assessments have yielded similar findings (Hofmann et al., 2013; Becker et al., 2019), underscoring the complexity of emotions associated with self-control challenges.

Though studies have employed a wide variety of research designs to examine the association between exerting self-control and the emotions that follow successes and failures, to our knowledge, no studies to date have attempted to offer a qualitative analysis of the emotional experiences resulting from self-control dilemmas. The main limitation of this dearth of qualitative work is that quantitative studies of self-control and emotions have overwhelmingly focused on conflicts and domains pre-selected by researchers, often without consideration of whether a given conflict is of relevance to participants. Relatedly, most studies to date have assumed that a given outcome (e.g., exercising, eating healthily) is a self-control success for all participants, a methodological choice that can overlook idiosyncratic goals and the complexity of self-control experiences. For instance, though exercise may be a goal-directed behavior that requires self-control to enact, for some people (e.g., a runner rehabbing an injury) or in some situations (e.g., when one has a paper deadline to attend to), exercising may not be a goal-consistent behavior. And while both categories of experiences may engender negative emotions (i.e., not exercising due to laziness, not exercising due to injury or conflicting goals), they may entail entirely different experiences. One notable exception in this approach is an experience sampling survey by Hoffman et al. (2018) in which participants could select from dozens of desires and conflicting goals for dilemmas that they experienced throughout the day, over the course of 7 days.

The purpose of the present study is to extend this growing literature by qualitatively analyzing open-ended descriptions of self-control conflicts and the emotions that accompanied self-control successes and failures. We extend the extant literature in at least two ways: First, we do not impose restrictions on the domains of self-control dilemmas participants report experiencing, allowing participants to describe situations that were salient and relevant to them. Second, we use open-ended responses in an effort to avoid cuing specific goals or emotions in response to self-control dilemmas and outcomes. This approach allowed participants to describe any type, number, and progression of emotional experiences, potentially providing a more complex and detailed picture of the emotions that accompany self-control conflicts. To better understand how these emotional experiences may vary as a function of the type of self-control demands, self-control dilemmas were divided between those that require inhibition and those that require initiation of behavior, a distinction that has been largely ignored in prior work on emotions associated with self-control.

Our study had two objectives. The first objective was to examine variability in participants’ experiences that required self-control, uncovering types of concerns generally relevant to participants and where, with whom, and when these concerns typically took place. Second, we aimed to examine the emotions that followed successes and failures of self-control of two types: initiation and inhibition. By pursuing these two objectives, we hoped to gain an understanding of the naturally-occurring complexity of self-control dilemmas and how those emotional experiences may differ depending on the type of self-control they accompany.

## Materials and Methods

### Overview

This study took a qualitative approach to the examine the patterns of emotional experiences in the context of self-control dilemmas. The survey was distributed on MTurk, a crowdsourcing online platform that provides research access to diverse and representative samples of Internet users, called workers (Buhrmester et al., 2011). Through the platform, workers can view all studies for which they are eligible to participate (called Human Intelligence Tasks; HITs), as well as the pay, and select which to join. The compensation for participation is directly transferred to the participants’ Amazon accounts.

Workers residing in the United States provided short descriptions of recent self-control dilemmas and their emotional experiences during and after successfully or unsuccessfully meeting self-control challenges. All study procedures were approved by the authors’ institution’s ethical review board. Responses were organized into individual transcripts by the primary investigator, and coded by the first author using NVivo software (v12, QSR International Pty Ltd., 2020).

### Participants

Participants were 187 MTurk workers recruited through the MTurk platform in December 2010, and paid $0.50 for a 1-h survey. Participants were eligible to participate if they lived in the United States and had at least 95% approval rate. For the purpose of the present analysis, only participants who completed both the initiation and inhibition portions of the survey were included in the analysis (*N* = 132 participants, 70.59%), for a total of 528 coded descriptions of self-control dilemmas. Participants were between 18 and 72 years old (*M* = 33.27, SD = 11.96, 71% female) and represented multiple occupations (20% were students), including truck driver and botanist.

### Procedures

MTurk workers were informed that this study “involves reflecting on and describing decisions you made about your own behavior from the past 2 weeks.” The survey was structured in four blocks: two for self-control dilemmas that required behavioral inhibition, and two for dilemmas that required behavioral initiation. Within each block, one set of open-ended items referred to a dilemma in which the participant successfully exercised self-control and another set that referred to a dilemma in which the participant failed to exercise self-control. Supplementary Tables 1, 2 outline all question prompts. Inhibition prompts were always presented before initiation prompts. Data and all materials are available at https://osf.io/khb6e/.

Each set of questions began by instructing participants to recall a situation in which they had an impulse to behave in a way they should not or to not behave in a way they should given their goals or preferences (i.e., they experienced a self-control dilemma). For instance, in prompting participants to recall a situation in which they needed to inhibit a temptation and they *successfully* stopped themselves, the prompt read “*vividly recall a situation in which your impulse was to behave in a particular way, but you thought you should not and successfully stopped yourself from doing so.*” In prompting participants to recall a situation in which they successfully initiated a behavior that they did not want to enact, the prompt read “*vividly recall a situation in which your impulse was to NOT behave in a particular way, but you thought you should and successfully made yourself do so.”*

Prompts that referred to failures—either to inhibit or initiate a behavior—replaced the term “*successfully [made yourself do so/stopped yourself from doing so]*” by “*unsuccessful [in making yourself do so/stopping yourself from doing so].*” Each potential combination of self-control type (inhibition, initiation) and outcome (success, failure) composed one of four prompts. All participants saw all four prompts, ultimately being asked to describe two instances of self-control by inhibition and two of self-control by initiation.

Participants were then asked to select from a drop-down menu how long ago the decision took place (ranging from 1 to 14 days, in increments of 1 day), and to respond to open-ended items asking them to describe the self-control dilemma, the type of behavior that it involved, the situation in which it occurred (e.g., who was with them), why they thought they were successful or unsuccessful in stopping themselves or making themselves act, their thoughts at the time, and emotional experiences during and after the decision. These items were repeated across the four blocks and modified to reflect the type of self-control required in each dilemma (i.e., inhibition, initiation), and the outcome of the dilemma (i.e., success, failure). Participants could write as much or as little as they wanted, and about any dilemma they desired. Analyses presented here focus on the type of impulse, the situation that surrounded it, the outcome, and the emotional experiences that followed the outcome.

### Analysis

Analyses began by attempting to identify patterns of responses that could inform the emotion-related themes for later coding. This process involved reading the first 31 cases in the dataset (23.5%) as well as one case selected for the richness of its descriptions. The latter case and its elaborate descriptions informed a document memo, whereas the former cases informed a reflection memo on the data. Both memos were integral to the initial understanding of the data, as they highlighted themes that could be more easily observed in complex descriptions, as well as commonly occurring themes and emotions.

Informed by the document and reflection memos, as well as repeated reading of the descriptions, the first author outlined the codebook, which was further refined after being applied to the first five cases in the dataset. Through this iterative process, new codes were generated to capture the themes in the data, and codes that did not refer to the main research question were removed. The final version of the codebook was then used to code all responses.

Not all emotions described by the participants were anticipated during the development of the codebook. Thus, at the time of formal coding, new emotions were added to the codebook if the current list of codebook emotions did not capture the information conveyed by participants. All responses were coded by the first author using NVivo software (v12, QSR International Pty Ltd., 2020). Data analysis was aided by composing a memo of the coded data and tables outlining the emotions following dilemmas.

For parsimony and interpretability, emotions mentioned by participants were collapsed into fewer categories based on semantic similarities. Table 1 summarizes the old and new categories, and Table 2 provides sample quotes for each emotion label. Specifically, across the sample of coded responses, participants described 35 different negative emotions and nine positive emotions *before* their self-control decision, and 25 different negative emotions and 16 positive emotions *after* their self-control decisions. This process reduced the emotion labels to 19 negative and eight positive emotions *before*, and 13 negative and nine positive emotions *after* self-control decisions. Emotions were analyzed separately, and only those described to occur *after* the self-control decision were included in the analyses presented here.

**TABLE 1:** Summary of changes to emotion labels.

Negative emotions		Positive emotions	
Aggregate label	Original label	Aggregate label	Original label
*Anxiety*	Anxiety, nervousnes overwhelmed, stress, and worry	*Accomplishment*	Accomplishment
*Anger*	Anger, annoyance, frustration, and irritation resentment	*Attraction/desire*	Sexual arousal, sexual attraction
*Apathy*	Apathy, boredom, disinterest, resignation, and somber	*Calm*	Calm
*Disgust*	Disgust	*Confidence*	Empowerment, determination, and righteousness
*Embarrassment*	Embarrassment	*Energetic*	Energetic and excited
*Fear*	Dread and fear	*Gratitude*	Gratitude
*Guilt*	Guilt	*Happiness*	Amusement, contentment, happiness, pleasure, refreshed, and satisfaction
*Jealousy*	Jealousy		
*Laziness*	Laziness	*Pride*	Pride
*Longing*	Longing	*Relief*	Relief
*Regret*	Regret		
*Sadness*	Agony, disappointment, sadness, and upset		
*Shame*	Shame		

**TABLE 2:** Sample quotes per emotion category.

Emotion	Sample quote
Anxiety	I experience nervousness [*sic*] and am paranoid. I felt that since i did not have any money to buy it i could steal it instead. So i suppose i felt greed [shoplifting; initiation, successful]
Anger	I was angry with myself all weekend over this. Just anger and resentment [physical aggression; inhibition, successful]
Apathy	The morning I woke up, I decided not to act on the impulse to cancel the interview. My dread and anxiousness faded, and I then felt a calm apathy toward the situation [skipping interview; inhibition, successful]
Disgust	I felt a little disgusted with myself for choosing the unhealthy dish [food; inhibition, unsuccessful]
Embarrassment	I was very angry and desperate when I threw the chair and felt ashamed of myself and embarrassed after I did it [physical aggression; inhibition, unsuccessful]
Fear	Fear of getting pulled over and getting a ticket [speeding; inhibition, unsuccessful]
Guilt	Extreme guilt. I only felt a little guilty at first, but when I had seconds, I was disgusted with myself [eating; inhibition, unsuccessful]
Jealousy	I felt jealously, anxiety, and tension during and after my decision [criticize someone; inhibition, successful]
Laziness	I felt guilty for not cleaning up and lazy for not doing my part. I also felt relaxed and had a great time on the couch all day [chores; initiation; unsuccessful]
Longing	Well, I really longed for that meal, but was proud to choose not to eat just to eat and to make alternate plans for later [eating; inhibition, successful]
Regret	(…) I felt happy for a minute after I bought some gifts but the next day I regretted my decision when the bill collector called me [shopping; initiation, successful]
Sadness	I felt oddly relieved to have decided not to buy the TV but at the same time I felt disappointed [shopping; inhibition, successful]
Shame	At the beginning I experience courage to think it was my time to shine but, after I didn’t step up I felt ashamed. [work; initiation, unsuccessful]
Accomplishment	I was upset and lethargic beforehand but felt accomplished afterward [chores; initiation, successful]
Attraction	Happy, anxious, a little nervous, sexual [social contact; inhibition, unsuccessful]
Calm	I think I felt a superficial calm and comfort, the narrow gratification of easing my discomfort and soothing myself in the moment [go to bed; initiation, unsuccessful]
Confidence	I experienced gratification I felt a little guilt but that was overcome with feeling it was the right thing to do [social contact; inhibition, unsuccessful]
Energetic	I was feeling excited and happy [shopping; inhibition, unsuccessful]
Gratitude	Honeslty [*sic*], having wonderful friends that care about you and are wiling to help you though anything. It made such a fearfull [*sic*] experience for me, now seem bearable [obtain service/self-care; initiation, successful]
Happiness	I was really happy after I bought it and also a little bit sad because now I don’t have any money at all [shopping; inhibition, unsuccessful]
Pride	During I felt mostly guilt for even thinking considering the impulse in the first place, but afterward I felt relief, and even pride, that I had not followed through with it [leisure; inhibition; successful]
Relief	The impulse was to leave work, knowing if stayed I could make some extra money. I left. I felt a sense of dread while there and a sense of relief as I left [work; initiation, unsuccessful]

To purpose of this analysis is to offer an account of the complexity of self-control experiences via descriptions of emergent themes. To help readers gauge differences between types of self-control and their outcomes, we also provide the prevalence of emotions in response to each of the four prompts. To compute prevalence (proportions), we counted emotions only once per prompt per participant (e.g., if a participant mentioned happiness twice in response to successful inhibition, happiness was counted only once for that prompt and participant). We then divided the number of times an emotion was mentioned in a prompt by the sum of all times all emotions were mentioned in that same prompt.

## Results

### Objective 1: Describing Self-Control Dilemmas

#### Challenges

Most impulses described by participants took place the day before the survey and 14 days prior (26% and 18%, respectively), potentially due to a combination of salience of events in memory for the more recent events and generalization of the date for the later events.

Approximately 65% of self-control challenges occurred while participants were in the presence of other people and 35% while participants were alone. As can be seen in Table 3, this was true across descriptions in self-control by inhibition and initiation, and across success and failure descriptions, though descriptions that involved failures of self-control by initiation were nearly evenly split between instances that occurred alone and with other people. In cases when participants were not alone, the situations were nearly equally divided between being in the presence of partners, children, co-workers, other family members, friends, and unknown others. Though being in the presence of unknown others occurred with greater frequency than with any other category of people, this was only marginally so. Congruent with this finding, about half of the challenges (51%) were described as occurring in places where unknown others may be present, such as during a commute, in public locations (e.g., bar, mall), school, and work.

**TABLE 3:** Frequency and percent of self-control challenges that occurred alone and not alone.

Situation	Inhibition		Initiation	
	Unsuccessful	Successful	Unsuccessful	Successful
Not alone	87 (66%)	84 (64%)	73 (55%)	84 (64%)
Alone	45 (34%)	48 (36%)	59 (45%)	48 (36%)

The most common challenge across all responses referred to social situations, appearing at least once for 85% of participants, and accounting for nearly half of all challenges described across participants. Supplementary Table 4 lists categories of coded challenges and sample quotes. Of the social self-control challenges, nearly two-thirds referred to antisocial desires, ranging from the desire to avoid social interactions at social events, avoid a phone call, or yell at someone, to the desire to engage in physical aggression against others (e.g., one’s children, partner, strangers). All prosocial challenges involved physically or emotionally approaching other people, such as “wanting to talk to a woman,” “call up a friend,” give advice to a friend or family member, and wanting to kiss one’s partner in public. Interestingly, a number of prosocial impulses were accompanied by antisocial behaviors that functioned as mechanisms for enacting the prosocial impulse, including one participant who described wanting to help out a friend by lying to someone else. Such occurrences underscore the complexity of self-control challenges, pointing to instances in which a traditionally “denounced” behavior (i.e., lying) was not undesirable to the participant; rather, in the situation, it was functional to achieve another goal (i.e., help a friend).

The second and third most common impulses related to food, such as eating sweets or eating when not hungry, and work or school activities, such as wanting to skip classwork or not go on a business trip. Spending money on shopping was also a commonly described impulse, ranging from household goods such as a vacuum cleaner and storage bins, to personal items such as a purse, a fishing reel, and food items. Though the majority of shopping self-control challenges referred to items for oneself, a subset referred to “prosocial” shopping, such as purchasing Christmas gifts for one’s family or charity. A less frequently-described challenge in the domain of shopping and finances referred to behaviors aimed at saving or making money. Such impulses included wanting to borrow money from one’s parents, skipping a bill payment, making adult videos for supplemental income, and not wanting to drive their child to an event in order to save fuel.

A small portion of self-control challenges referred to behaviors aimed at expressing or avoiding the expression of emotions and needs. This category involved relatively harmless behaviors such as crying even though one did not want to cry and expressing frustration in a letter to the local newspaper. However, it also involved personally harmful behaviors such as avoiding pursuit of medical care due to fear and hurting oneself or committing suicide.

#### Initiation Versus Inhibition

Dilemmas that involved inhibition of impulses spanned all challenge categories. Some examples were eating junk food and sweets (e.g., chocolate, ice cream, and cookies), cursing at someone, “taking time off work to have fun,” physically chastising one’s child, cheating on a partner, sleeping in, and using drugs. Notably, the only instance in which a chore required inhibition was a parent’s description of wanting to “give in to [their] daughter’s demand to stop by her school after cheer practice to see the last 15 min of a science and engineering event.”

Self-control challenges that required initiation spanned fewer categories than those requiring inhibition. For example, no participants described wanting to initiate deviant behavior (e.g., lying) or aggressive behavior (e.g., punching someone) but struggling to do so. Common challenges that referred to initiation pertained to chores (e.g., not cleaning the kitchen), positive social encounters (e.g., initiating a positive conversation with the mailman; leaving on time for a family commitment), and caring for oneself (i.e., rewarding oneself with a treat). The mention of rewarding oneself for a behavior that requires effortful initiation rather than inhibition underscores the contention that challenges typically associated with inhibition may not be ideal to all. Indeed, whereas rewarding oneself required inhibition for some participants; for others, it required initiation to overcome other salient goals, like saving money. This pattern raises important considerations for quantitative studies, in which the—albeit reasonable—assumption that some behaviors are generally undesirable could lead to the underestimation of important effects.

Emotions associated with self-control challenges were often accompanied by quantifiers about the intensity of the experience. These were especially common in the case of inhibition failures and in relation to negative emotions. Qualifying adjectives indicating low levels of emotions were most common, followed by adjectives indicating intense emotions, such as “really,” “extreme(ly),” and “intense.”

Across all challenges, three prominent themes emerged. The first two themes pertained to the content of self-control dilemmas, defying the understanding that successful self-control is an asset. Specifically, self-control successes did not necessarily involve conventionally “good” behaviors, and self-control failures did not involve conventionally “bad” behaviors. That is, self-control successes could have involved behaviors typically seen as harmful, such as successfully engaging in substance use despite not wanting to. Likewise, self-control failures could involve behaviors that posed no harm or were even arguably beneficial, such as sticking to one’s diet and failing to eat desserts despite planning to do so during the holidays. The third prominent theme across the descriptions was participants’ struggle to recall dilemmas that required solely inhibition or initiation of behavior. Each of these themes is described in more detail in the following sections.

##### Self-Control Success Did Not Necessarily Involve Engaging in “Good Behaviors”

Though most participants’ descriptions of successful self-control entailed reaching, or moving closer, to positive goals, this was not the case for all participants. One participant, for instance, described smoking marijuana to be a behavior that required self-control by initiation, “I did not want to smoke in case I was tested because that would break my parole. However, I wanted to in order to celebrate his birthday with everyone.” In this participant’s description, successful self-control by initiation would have involved substance use, a behavior that could have had tangible consequences for them (i.e., breaking parole). Note that, without context, this description could have been interpreted as a case of unsuccessful inhibition of the impulse to use substances. However, the participant later described caring about what others thought of them and about not letting others down, suggesting that smoking marijuana was not an impulse to be overcome. Rather, it was the primary goal for the purpose of maintaining social relationships.

A similar case was a participant who described wanting to hang out with friends, who in turn wanted to drink more than the participant was “comfortable with.” The participant described participating in the drinking behavior anyway because they wanted to spend time with the friends. Another participant described struggling over whether to reply to a Christmas card from a person they did not like, weighing in their own desire not to engage further and their desire not to “hurt” the other person. The participant described successfully inhibiting their desire to reply to the card, a behavior that may ordinarily be perceived as antisocial but that was—in this case—helpful for the participant, who described feeling relieved in turn.

##### Self-Control Failures Did Not Necessarily Involve Engaging in “Bad Behaviors”

In the same vein, though most participants’ descriptions of unsuccessful self-control involved enacting negative or generally undesirable behaviors, self-control failures often involved enactment of generally good or desirable behaviors. For example, one participant described failing to resist the urge to clean their room before the date they stipulated for themselves, a personally undesirable behavior because they would need to spend money on storage bins. Another participant described having a goal of “forgetting the diet, and eating all [they] wanted. Seconds, thirds and desserts.” However, they failed to initiate this behavior and instead, watched their calories and did not have dessert. Of note, though the outcomes of these participants’ experiences were not negative on their own (i.e., cleaning, sticking to a diet), these participants reported anticipated or actual consequences of their failures, such as expecting to feel disgusted with themselves and spending money to buy storage bins.

##### Difficulty Separating Initiation and Inhibition

This theme was readily apparent in many descriptions and involved situations in which enacting one form of self-control also involved enacting a second, such as initiating one behavior while inhibiting another. In one case, a parent described wanting to give in to the impulse to drive out of their way and “give in to [their] daughter’s demand to stop by her school after cheer practice.” In this instance, the parent not only described the need to initiate a personally difficult behavior (i.e., driving out of their way to satisfy their daughter’s demand), but also to inhibit an impulse (i.e., “give in to my daughter’s demand”). Ultimately, the parent described following the impulse to “give in” because they did not want to “fight with [their daughter] anymore.” This description suggests that self-control is not an either-or process, whereby people either do or do not engage in self-control. Rather, as was apparent in descriptions like this one, it seems that participants balanced multiple goals at once. In the case above, it appears that the parent’s decision to give in to their daughter was not a simple decision between “to give in or not to give in”; but between giving in, going out of their way home, and ending an argument, or resisting the temptation to give in, continuing the ride, and enduring an argument. Such experiences raise questions about assumptions that specific behaviors (e.g., giving in to others) are evidence of lack of self-control, a common assumption in quantitative studies of self-control. Rather, in some cases, they may entail a complex process in which different forms of self-control are necessary, multiple goals are salient, and other self-regulatory processes are at play, ultimately leading to self-control decisions in which one goal is prioritized over the others.

Though participants were prompted to describe challenges that required inhibition with the same frequency with which they were prompted to describe challenges that required initiation, challenges by inhibition were more commonly described than those by initiation. That is, in many cases, when participants were prompted to describe a situation that required initiation, they instead provided a situation that required inhibition. Even among cases in which participants described a situation that required initiation of behavior, the same situation also required inhibition of an impulse. In one such example, a participant described needing to cancel a doctor’s appointment the next day in order to sleep longer hours. Such conflict is both one of failing to inhibit the impulse to cancel a doctor’s appointment and, ultimately, an example of failing to initiate the behavior of going to the doctor.

In a similar example, a participant described having the impulse to write “a hateful letter” to the developer of a new park and marina near the participant’s house. Not only did the participant report not sending the hateful letter, but instead “writing a sensible one espousing both the good and the bad about the developing park and marina.” In this case, the participant exercised self-control by both inhibiting the impulse to write an angry letter and initiating the behavior of writing a kind letter despite their original impulse. Similar cases were described by other participants in other contexts, such as successfully inhibiting the impulse to eat a cookie and, instead, successfully initiating the behavior of eating an apple.

Interestingly, whereas instances of pure initiation were rare and self-control by inhibition was frequently mentioned in cases that required self-control by initiation, most descriptions of self-control by inhibition were purely so. That is, descriptions of self-control by inhibition (both successes and failures) did not often involve replacing the inhibited behavior with the initiation of another (e.g., not punching someone). On the other hand, instances that demanded self-control by initiation frequently involved inhibition of the alternative impulse (e.g., remaining calm and not yelling at someone else).

### Objective 2: The Emotions Surrounding Self-Control Outcomes

Overall, positive emotions were described less frequently (42% of all instances) than negative emotions (58% of all instances), both in number of distinct labels used (e.g., happy, calm, accomplished) and in number of instances across all narratives. Although positive emotions were more frequent than negative ones in response to successes of inhibition (55% of all mentions) and initiation (60% of all mentions), they comprised 28% of all mentions in response to inhibition failures and 23% of all mentions in response to initiation failures.

#### Inhibition

Inhibition responses were complex and rich. Many participants wrote long paragraphs, many of which included complex descriptions of situations that involved both initiation and inhibition of behavior, multiple goals, and multiple emotions. For example,

*I entered my daughter’s room, she lives at home, and immediately was met with the aroma of old food, stale cigarettes and gross perfume. Because I had previously chosen not to discuss the state of HER room, I said nothing about the room, although inside I was really angry at the clear lack of respect for the house we provide for her to live in. What I wanted to talk about was her recent decision to move out of state and live with her boyfriend. I set the tone by telling her that I hoped we could talk calmly and openly as adults about some of the specifics of her plan. Well after about 1 minute things turned ugly and she started laughing at my attempts to talk about things like money and jobs. So I lost my temper and shouted at her which I had tried so hard not to do.*

In this response, the parent describes having a goal to initiate a calm and open conversation, which ultimately not only failed, but was followed by “I lost my temper and shouted at her,” which the participant explicitly described wanting to prevent. From the description, it appears the participant had multiple goals, from talking to their daughter about moving out, to inhibiting the impulse to talk about the state of the room. In response to the altercation, the participant described, “I experienced anger, hurt, disappointment, frustration and guilt while I was shouting and after I shouted,” providing one example of the variety of emotions that typically followed inhibition-related responses. Despite the complexity apparent in the inhibition responses, four themes consistently emerged throughout participants’ experiences, two in reference to inhibition successes and two in reference to inhibition failures. In this section, these four themes are described along with examples from different participants.

##### Inhibition Failures Can Feel Good

By definition, Failing to exercise self-control by inhibition often entails engaging in a desired behavior that conflicts with another goal. As such, it is perhaps unsurprising that many participants’ self-reported failures to inhibit behaviors were met with positive emotions. In fact, positive emotions accounted for 28% of the emotions reported in response to inhibition failures. Happiness was among the most common emotions, comprising 18% of all mentions. For comparison, the most common negative emotion, guilt, made up 16% of all mentions in response to inhibition failures. Positive emotions emerged in response to inhibition failures even when participants acknowledged having goals that conflicted with their behavior. For instance, one participant described feeling happy, powerful, and “not remorseful,” after cheating on their partner, even though the participant also described loving them.

Notably, however, for most participants who reported positive emotions after inhibition failures, their experiences did not end with positive emotions. Rather, positive emotions were frequently followed by negative emotions. One example is a participant who described giving in to the impulse to gossip about another person even though they knew they should not. As was the case with previously described experiences, the participant reported positive feelings of elation, however, these were followed by guilt and anger at themselves. Experiences such as this were common across descriptions. Other participants described feeling good then regretful after eating past fullness, whereas others described relaxation followed by disappointment in themselves for drinking alcohol. Importantly, the pattern of positive-then-negative emotions also accompanied descriptions of successful inhibition. However, in this case, it was usually followed by relief then a negative emotion such as guilt for having the impulse and regret for not engaging in the desirable behavior. One such example is a participant who described successfully inhibiting their impulse to hit a friend. Though the participant described feeling relief at their success, they also described feeling guilty at having had the impulse. Likewise, another described feeling relieved that they resisted the impulse to eat something tasty yet unhealthy, but this feeling was accompanied by regret.

##### Anger, Guilt, Regret, and Shame Were Common After Inhibition Failures

As evidenced by the cases described above, inhibition failures were frequently followed by descriptions of guilt (16%), regret (15%), anger (14%), and shame (4%). Anger, the only emotion in this subset typically categorized as non-self-conscious, was often described to be aimed at oneself—though less frequently so than toward others.

Descriptions of guilt and shame were common across all inhibition failures, including failure to inhibit the impulse to overeat, and particularly common when failures involved social situations. For example, in describing an instance of failure to inhibit an impulse, one participant described wanting to throw a book across the room to get their boyfriend’s attention during an argument. In addition to feeling angry at the outcome—also a common emotion in response to inhibition failures—the participant described feeling embarrassed by their own behavior. Likewise, a parent described feeling “horrible and guilty” after yelling at their daughter, whereas another participant described feeling “slightly guilty” after lying to a friend to avoid going out. The frequency with which guilt appeared in response to failures of self-control that involved social situations is consistent with the notion that guilt—as well as shame and embarrassment—are self-conscious emotions, which are of particular importance for social outcomes (Tangney and Tracy, 2012).

In some cases, guilt was not a consequence of the behavior itself (e.g., yelling at someone) but of an outcome of the behavior. One participant, for example, described having been “verbally inappropriate” with their boss by having sworn and “attacked some of her recent decisions.” In describing their emotions after the choice not to “keep my mouth shut to save my job,” the participant described feeling regret because their outburst cost them their job, as well as guilty because the job loss had repercussions for their ability to support their family. In a similar case, a participant described failing to inhibit the impulse to spend their Christmas bonus at the casino, and feeling “depressed and sad since losing the money” rather than sad about the choice to go to the casino. That is, unlike cases in which negative emotions arose in response to the decision itself (e.g., guilt at yelling at one’s child), a smaller number of participants reported experiencing negative emotions in response to the *consequences* of their choices.

##### Inhibition Successes Can Feel Like Unresolved Temptations

Though successful inhibition of a given behavior brings one closer to another goal, these other goals are often far in the future or not easily observed. For example, whereas eating chocolate and breaking a diet can be immediately pleasurable, resisting the urge to eat chocolate and sticking to a diet may not yield readily observable outcomes. This means that successfully inhibiting a behavior may leave one without immediate rewards and, instead, with what appears to be unresolved temptations. For instance, one participant described feeling relief that they succeeded at inhibiting the impulse to go to work when sick, but regret because they “needed the money quite a bit.” Likewise, a participant described successfully inhibiting the impulse to be rude to their boyfriend’s father even though they did not truly “count it a success.” Rather, the participant described the experience as an instance in which “[the participant’s] “good manners” won out over [the participant’s] bruised moral compass.”

##### Inhibition Successes Can Also Feel Relieving

As noted by the recently-described participant who felt relief that they were able to inhibit their impulse to go to work when sick, relief accounted for nearly one-third of all positive emotions and 16% of all emotions (positive and negative) in descriptions of inhibition successes. Relief was especially common in the context of relief that “the alternative” did not happen. For example, upon successfully inhibiting the impulse to hit someone, a participant described, “I experienced intense anger during the experience, but relief that I had not bloodied [my] fist on such a petty individual afterward.” Similarly, one participant described feeling relief that they complied with their parent’s request not to go to a party, as disagreeing would have potentially jeopardized their fragile relationship.

Though relief was mainly reported in response to inhibited social impulses (both prosocial and antisocial), it followed successful inhibition of other impulses as well. One participant, for example, described relief that they did not stop by a fast-food restaurant on their way home, whereas another described feeling relief that they would not have to worry about academic dishonesty because they successfully inhibited their impulse to cheat. A third participant described feeling relief that they inhibited their impulse to buy an expensive purse, whereas a fourth described relief at not drinking due to the potential for automobile accidents.

#### Initiation

Initiation responses were more limited in content (i.e., shorter descriptions) than inhibition ones, potentially because initiation prompts consistently appeared after inhibition ones. Moreover, initiation failures, in particular, may be less noticeable than inhibition failures, as the outcome is the less-observable absence of a behavior. Nevertheless, two main themes emerged in relation to the descriptions of self-control by initiation. The first was that initiation success was generally associated with positive emotions, and the second was that initiation failures tended to be accompanied by anger, regret and guilt.

##### Initiation Successes Felt Good

For example, one participant described being strongly agoraphobic yet wanting to go to a city council meeting to argue for an issue of importance to them. Though the participant described feeling anxious before and after the meeting, they also reported feeling good about themselves for their success. Another participant described feeling stressed and sad that they could not continue watching movies into the night, but that they felt refreshed after going to bed. Other instances of positive emotions in response to initiation success were feeling good at having initiated a positive conversation with someone, pride for choosing a healthier food option, relief after engaging in a dreaded exercise session, and both relief and happiness at initiating a difficult conversation with one’s boss.

Overall, positive emotions comprised 60% of all mentions in response to successful initiation prompts. Happiness accounted for more than half (54%) of all positive emotions, followed by relief (24%), confidence, pride, and calm (all 6%).

##### Initiation Failures Were Mainly Accompanied by Anger and Guilt

As was the case with inhibition failures, initiation failures were mainly accompanied by feelings of anger (13%) and guilt (26%). One such example was a participant who described failing to return a call from their sister, who had asked for help from the participant’s husband, who fixes computers. Though the participant described feeling annoyed and unmotivated to return the call, they also described feeling guilty for acting on their impulse. Similarly, though a participant wrote “…who cares? Will do it later” in response to “putting it off” making a necklace for her co-worker, the same participant reported “still feeling a little guilt” for their failure to initiate the behavior she intended. Regret (9%) and shame (4%) were also mentioned in response to initiation failures. Note that whereas regret was less-commonly listed after failures to initiate behaviors than failures to inhibit them, guilt was more prevalent in the former than the latter. These differences suggest that failures in each type of self-control may be associated with different emotion signatures.

## Discussion

This qualitative analysis examined the complexity of goals that give rise to self-control challenges and the emotional experiences that follow dilemmas requiring initiation or inhibition. Overall, the self-control dilemmas described by participants ranged from simple everyday decisions like whether to eat dessert, to more complex descriptions of self-control conflicts, like feeling compelled to drink in order to socialize despite not wanting to drink, and management of multiple goals, like venting frustrations to others while maintaining positive social relationships. Across scenarios, descriptions of emotional experiences ranged from simple labels to complex narratives about changes in valence and intensity (e.g., feeling happy about indulging, then guilty about it). Importantly, this qualitative analysis revealed a sometimes blurred line between self-control by inhibition and initiation, and a variety of goals that challenge the constrained set of dilemmas commonly taken to require self-control.

Across dilemmas of inhibition and initiation, successes and failures, *social challenges—*both prosocial and antisocial—were the most common challenges described by participants. This prevalence is likely due to the relevance and pervasiveness of social relations in daily life, from family and friends to co-workers and strangers (Baumeister and Leary, 1995). Notably, the high prevalence of social-related challenges differs from prior work showing that social desires (e.g., desire for social contact) were far less common than food-related desires (Hofmann et al., 2012). One possible explanation for this difference is that *antisocial* impulses were underreported in previous work due to social desirability, multiple-choice questionnaires, or simply the timespan of interest to researchers. By using an open-ended format and allowing participants to report any experience up to 14 days before the survey, participants were not constrained to any type of challenge or to short timespans, allowing them to report any personally meaningful self-control challenge even if it occurred 2 weeks before the study.

In the present sample, *negative emotions were more varied* than positive emotions, and mentioned more frequently than positive emotions across all types of narratives. Of note, although the outcome of self-control successes involved mainly positive emotions like relief, happiness, and pride, negative emotions were reported in response to both successes and failures of self-control. The high prevalence of negative emotions following self-control successes and failures may stem from how memory is encoded for emotional events. Whereas emotional memories are generally encoded with more detail than neutral memories, memories associated with negative emotions are better recalled by both younger and older adults, whereas memories associated with positive emotions are better recalled by older adults only—compared to neutral memories (Kensinger et al., 2007). Our sample was, on average, composed of young adults, potentially explaining the higher prevalence of negative emotions over positive ones.

Among the negative emotions commonly-described, *guilt* was the most prevalent emotion in response to failures of both types of self-control. Notably, this was the case even though participants did not receive emotion prompts and generated emotion labels themselves, potentially due to the relevance of emotions like guilt, shame, and pride for the self and social relations (Tangney, 1999; Tangney and Tracy, 2012). Unlike failures to initiate behavior, which are more consistent with missed opportunities to act, failures to inhibit behaviors often entail observable incongruencies between behaviors and self-relevant goals and values. Such failed inhibition and what it may signal to oneself and others could engender feelings of guilt in ways that differ from failed initiation, as the outcome of the latter is the less observable absence of a desired behavior.

Interestingly, although guilt was more prevalent in failed initiation scenarios, regret was most prevalent in inhibition ones. These potentially different emotional signatures of failures of initiation and inhibition may indicate that greater awareness or sensitivity to either emotion could be related to different behavioral propensities.

Consistent with work showing that anticipated guilt can foster exertion of self-control (e.g., Kotabe et al., 2019), guilt was also mentioned when participants described the emotions that arose when thinking of the challenge, even if they successfully met it. Guilt was also described to follow *consequences* of self-control failures, and not the self-control failure itself, suggesting that research on the emotional consequences of behaviors may benefit from longer-term assessments to fully capture the emotional consequences of self-control dilemmas.

In comparison, *relief* was a common emotion after inhibition successes, such as relief at complying with parent’s request not to go to a party, as disagreeing would have potentially jeopardized a fragile relationship; at not being rude at the veterinary clinic staff due to being seen late on a day the participant perceived the clinic to be busy; and relief that the participant did not respond angrily toward their partner even though they were upset at their partner.

### Limitations and Future Directions

The findings in this study should be interpreted within the scope of the present sample and design. Participants were a sample of MTurk workers who, although more diverse than college student samples (Buhrmester et al., 2011), are nevertheless unlikely representative of the general U.S. population. Second, the time in which the study was conducted (i.e., December) appears to have influenced a subset of participants’ responses in domains like shopping. Specifically, though most shopping-related challenges referred to personal purchases, some referred to Christmas gift shopping, suggesting that the season in which the data were collected may have influenced the considerations of a portion of the present sample (i.e., shopping for prosocial purposes like gift giving).

This pattern underscores future studies’ need to consider contextual information when examining participants’ self-control challenges, as the source of the sample or season in which the study takes place could inflate or underestimate the types of challenges most relevant to a given sample. For instance, it is possible that self-control failures at times when personal goals are highly salient (e.g., shortly after New Year’s Day) relate to more negative self-conscious emotions (e.g., shame, guilt) than at times when pro-social goals are more salient (e.g., before Christmas and other gift-giving holidays).

Future work may also investigate whether the less-usual dilemmas observed here would be deemed to require self-control in other samples. For instance, quantitative studies could examine participants’ ratings of the extent to which challenges involving socially-undesirable or illegal behavior require self-control. Because self-control is closely related to morality (Hoffman et al., 2018), it is possible that participants would not readily deem initiating illegal behavior to require self-control. Yet, these ratings may change if participants are provided with a definition of self-control as a goal conflict.

In cases where it was unclear whether participants were speaking about a failure to begin something or to stop themselves from doing something, due to the method of the survey, it was not possible to reliably determine to what the participants referred. For instance, in a successful initiation request, a participant wrote that their impulse was “To not tell a friend that they suck at being a parent. Not sure on what you mean by behavior as it was a general conversation on the phone.” In this case, it could be that the situation required telling someone something and the conflicting goal not to do so (i.e., requiring initiation); but it could also be that the impulse was to tell and the conflicting goal was not to say anything (i.e., requiring inhibition). Without more context from the participant, which the questions could not bring about effectively at times, it was not always possible to determine from what perspective participants were discussing their experiences. Future research using methods similar to those presented here should request additional descriptive information focused on features of the context relevant for understanding the nature of the self-control challenge.

In some cases, additional contextual information was not available until the end of the survey, when, unprompted, participants would describe not engaging or engaging in a behavior. However, this was not always the case. The same limitation was present in descriptions of emotional experiences, as the prompt asked for both emotions experienced *during* and *after* the decision. Without additional context offered by the participants, it was sometimes impossible to determine to what participants referred when they listed emotions without further context.

Finally, not all of participants’ descriptions appeared to refer to a self-control challenge. This was evident in descriptions that did not involve a conflict, either because the participant reported there being “no thinking involved,” or because the participants indicated not wanting to inhibit the behavior. Regarding the latter, one participant replied, “I wasn’t trying to stop myself from speeding, I just knew I shouldn’t be doing it because it can lead to a ticket. If I didn’t want to speed, I wouldn’t speed.” To the extent that self-control is a conscious choice between two or more goals, the absence of a conflicting goal or awareness of a conflict suggest that self-control was not required in such situations. Cases like this raise questions about the idiosyncrasy of self-control challenges, and speak to the value of considering participants’ macro- (e.g., social norms, culture) and micro-environments (e.g., mood, physiological states, memories, past experiences) in interpreting their responses.

From a broader self-regulation perspective, self-control failures may be consistent with good self-regulation if “temptations” support other long-term goals, such as maintaining social connections, enjoying food, or having fun. Longitudinal studies may address this question by examining whether poor self-control that supports other valued goals relates to more positive emotions and, ultimately, better psychological well-being. The opposite may be true of good self-control, especially if successes contribute to a narrow set of goals or detract from other valued goals.

### Contributions

The online nature of this survey allowed disclosure of a wide variety of behaviors, such as physical aggression against pets, friends, and children, displaced aggression in the form of breaking objects; use of illicit substances; and dishonest behavior such as shoplifting, using someone else’s credit card for personal shopping, and cheating. One participant described weighing their behaviors by whether it would break their parole or not. The mundane and often unusual situations described by participants suggest that self-control dilemmas are more varied than what studies typically investigate, indicating that allowing participants to describe personally relevant dilemmas may uncover stronger associations between self-control and behavior than currently observed.

The present study underscores the complexity of self-control challenges, evidenced by how frequently participants described situations that required both initiation and inhibition of behaviors as well as mixed emotions in response to outcomes. It also highlights the distinctions and apparent emotional profiles characteristic of self-control dilemmas involving inhibition or initiation, suggesting that these two forms of self-control are not only theoretically but also experientially distinct. Although qualitative work composes a very small proportion of studies in psychology, this methodological choice allows the emergence of important trends rather than assumptions about them. It also demonstrates that an understanding of self-control challenges and how they are met requires attention to the specific goals at play in a given situation. Ultimately, as evidenced here, no behavior that is typically cast as an indication of self-control requires self-control of all people all of the time.

This study uses qualitative responses to offer detailed evidence of the interplay between self-control and other self-regulatory processes in navigating personal goals and external demands in daily life—both of which are frequently at play in daily life. Unlike the approach that socially-desirable behaviors are universally desired (e.g., eating healthily, maintaining social relationships), the present study showed that even apparent failures of self-control (e.g., eating unhealthfully, avoiding social relations) were in service of other goals that supported participants’ overall well-being. That is, self-control may not be a process that is easily isolated through cognitive or decision-making tasks that pit one response against another. This study suggests that both positive and negative emotions follow successes and failures of self-control, and that not all successful self-control is good and not all unsuccessful self-control is bad. Most importantly, both can entail good or bad outcomes that cannot be easily parsed without detailed consideration of personal experiences and goals of participants.

## Data Availability Statement

The datasets presented in this study can be found in online repositories. The names of the repository/repositories and accession number(s) can be found below: Open Science Framework (www.osf.io/KHB6E).

## Ethics Statement

The studies involving human participants were reviewed and approved by Duke University Campus Institutional Review Board. The patients/participants provided their online informed consent to participate in this study.

## Author Contributions

RH: designed and executed the study, advised, and co-wrote the manuscript. FA: data analysis, primary writing, and conceptualization of the manuscript. Both authors contributed to the article and approved the submitted version.

## Conflict of Interest

The authors declare that the research was conducted in the absence of any commercial or financial relationships that could be construed as a potential conflict of interest.

## Publisher’s Note

All claims expressed in this article are solely those of the authors and do not necessarily represent those of their affiliated organizations, or those of the publisher, the editors and the reviewers. Any product that may be evaluated in this article, or claim that may be made by its manufacturer, is not guaranteed or endorsed by the publisher.
