# Supplementary material for: The Experience of Succeeding and Failing at Self-Control: A Qualitative Analysis
Source: Front Psychol. 2022 Apr 6;13:774059. doi: 10.3389/fpsyg.2022.774059 (PMC9019582; doi:10.3389/fpsyg.2022.774059)
Supplement: Supplementary file 1 [file Data_Sheet_1.pdf]

frontiers

Supplementary Material

1 Supplementary Tables

1.1 Supplementary Table 1

Question Prompts for Items About Self-Control by Inhibition

**Unsuccessful**
**Instructions:** Vividly recall a situation in which your impulse was to behave in a particular way, you thought you should not, but you were unsuccessful in stopping yourself from doing so.
About how long ago did the decision you have brought to mind take place? (drop-down menu, possible responses ranged from 1 day to 30 days).
Describe the impulse. What type of behavior did it involve?
Describe the situation. For example, where were you when you felt the impulse? Why you think you felt it then and there? Who was present?
As best as you can remember, what were you thinking while experiencing the impulse and deciding whether to act on it?
What emotions did you experience during and after your decision to act on the impulse?
Why do you think you were not successful in stopping yourself from acting on the impulse? What factors contributed to your lack of success?
**Successful**
**Instructions:** Vividly recall a situation in which your impulse was to behave in a particular way, but you thought you should not and successfully stopped yourself from doing so.
About how long ago did the decision you have brought to mind take place?
Describe the impulse. What type of behavior did it involve?
Describe the situation. For example, where were you when you felt the impulse? Why you think you felt it then and there? Who was present?
As best as you can remember, what were you thinking while experiencing the impulse and deciding whether to act on it?
What emotions did you experience during and after your decision to not act on the impulse?
Why do you think you were successful in stopping yourself from acting on the impulse? What factors contributed to your success?

1.2 Supplementary Table 2

Question Prompts for Items About Self-Control by Initiation

**Unsuccessful**
**Instructions:** Now, vividly recall a situation in which your impulse was to NOT behave in a particular way and, though you thought you should, you were unsuccessful in making yourself do so.
About how long ago did the decision you have brought to mind take place?
Describe the impulse. What type of behavior did it involve?
Describe the situation. For example, where were you when you felt the impulse? Why you think you felt it then and there? Who was present?
As best as you can remember, what were you thinking while experiencing the impulse and deciding whether to act on it?
What emotions did you experience during and after your decision to act on the impulse rather than override it?
Why do you think you were unsuccessful in making yourself act when your impulse was not to do so? What factors contributed to your lack of success?
**Successful**
**Instructions:** Vividly recall a situation in which your impulse was to NOT behave in a particular way, but you thought you should and successfully made yourself do so.
About how long ago did the decision you have brought to mind take place?
Describe the impulse. What type of behavior did it involve?
Describe the situation. For example, where were you when you felt the impulse? Why you think you felt it then and there? Who was present?
As best as you can remember, what were you thinking while experiencing the impulse and deciding whether to act on it?
What emotions did you experience during and after your decision to override the impulse not to behave?
Why do you think you were successful in making yourself act despite the impulse not to do so? What factors contributed to your success?

1.3 Supplemental Table 3

Summary of Changes to Emotion Labels

Aggregate Label	Original Label	Aggregate Label	Original Label
Anxiety	Anxiety, Nervousness Overwhelmed, Stress, Worry	*Accomplishment*	Accomplishment
Anger	Anger, Annoyance, Frustration, Irritation Resentment	*Attraction*	Sexual arousal, Sexual attraction
Apathy	Apathy, Boredom, Disinterest, Resignation, Somber	*Calm*	Calm
Disgust	Disgust		
Embarrassment	Embarrassment	*Energetic*	Energetic
Fear	Dread, Fear	*Gratitude*	Gratitude
Frustration	Frustration	*Happiness*	Amusement, Contentment, Happiness, Pleasure, Refreshed, Satisfaction
Guilt	Guilt	*Confidence*	Empowerment, Determination, Righteousness
Jealousy	Jealousy	*Pride*	Pride
Laziness	Laziness	*Relief*	Relief
Longing	Longing		
Regret	Regret		
Sadness	Agony, Disappointment, Sadness, Upset		
Shame	Shame		

1.4 Supplementary Table 4

Impulse Categories and Sample Quotes Per Category

Impulse type	Sample Quote
Chore	“The kitchen was a mess. There were no clean dishes but I didn't feel like cleaning anything.”<br>“Not wanting to go to another town to pick up a friend.”
Conform to Norm	“The impulse not to clap and be involved during a church service.”<br>“To laugh at a burial wake.”
Deviant Behavior	“Revenge, by slashing tires”<br>“To not follow the academic code of honesty and to check my answers for a take home test with the answers of one of my friends.”
Food	“I wanted to get fast food even though I was not hungry and had plenty of food at home. I wanted to binge eat.”<br>“I'm a chocolate addict and I desperately wanted a candy bar.”
Express Emotions or Need	“I wanted to write a Letter to the Editor of our local newspaper to complain about the decision made by the city council (...).”<br>“I felt like I needed to cry... but I did not want to. I couldn't stop myself.”
Physical Aggression	“I wanted to throw my son's television to the floor and destroy it.”<br>“My new neighbor blasts music in the morning. It's extremely irritating music. I wanted to scream and pound on the ceiling, but I didn't.”
Sleep and Leisure	“The impulse was to sit on the couch all day and do nothing.”<br>“I had a doctors (sic) appointment early in the morning and had a strong impulse the night before to cancel the appointment and sleep in.”
Social	“I wanted to yell at this contractor who was working on our bathroom. I wanted to give him a piece of my mind.” (antisocial)<br>“I had the impulse to send a message to someone in a virtual world, someone I have not seen online for a couple of years.” (prosocial)
Spending Money	“I'm short on money and didn't want to have to use up gasoline in driving 80 miles round trip”<br>“My impulse was to buy a new vacuum cleaner as my other one had broken.”
Substance Use	“The impulse was to smoke weed. This was a bad behavior because it involves drugs.”<br>“My impulse was to drop acid. I guess that's a bad behavior to do drugs, so I'm glad that I successfully stopped myself.”
Work and School	“It involved getting work done for college classes. My "natural" impulse was to slack off and perhaps neglect the work altogether (...)”<br>“I needed to email a colleague of mine in Indonesia about some collaborative work in the next year, but I was told that he would be away for a few months so I procrastinated doing this for a while”
